# Ultra-high dose rate FLASH irradiator at the radiological research accelerator facility

**DOI:** 10.1038/s41598-022-19211-7

**Published:** 2022-12-22

**Authors:** Guy Garty, Razib Obaid, Naresh Deoli, Ekaterina Royba, Yuewen Tan, Andrew D. Harken, David J. Brenner

**Affiliations:** 1 Radiological Research Accelerator Facility, Columbia University, 136 S. Broadway, Box 21, Irvington, NY 10533 USA; 2 Center for Radiological Research, Columbia University, New York, NY 10032 USA; 3 Present Address: SLAC National Accelerator Laboratory, Menlo Park, CA USA

**Keywords:** Radiotherapy, Techniques and instrumentation

## Abstract

The Radiological Research Accelerator Facility has modified a decommissioned Varian Clinac to deliver ultra-high dose rates: operating in 9 MeV electron mode (*FLASH* mode), samples can be irradiated at a Source-Surface Distance (SSD) of 20 cm at average dose rates of up to 600 Gy/s (3.3 Gy per 0.13 µs pulse, 180 pulses per second). In this mode multiple pulses are required for most irradiations. By modulating pulse repetition rate and irradiating at SSD = 171 cm, dose rates below 1 Gy/min can be achieved, allowing comparison of FLASH and conventional irradiations with the same beam. Operating in 6 MV photon mode, with the conversion target removed (*SuperFLASH* mode), samples are irradiated at higher dose rates (0.2–150 Gy per 5 µs pulse, 360 pulses per second) and most irradiations can be performed with a single very high dose rate pulse. In both modes we have seen the expected inverse relation between dose rate and irradiated area, with the highest dose rates obtained for beams with a FWHM of about 2 cm and ± 10% uniformity over 1 cm diameter. As an example of operation of the ultra-high dose rate FLASH irradiator, we present dose rate dependence of dicentric chromosome yields.

## Introduction

Dose rate effects have come under intense scrutiny over the last few years. In the context of *radiation oncology*, FLASH radiotherapy^1^ makes use of ultra-high dose rate (> 30 Gy/s) irradiations to reduce radiation effects in normal tissue, while providing the same level of tumor killing. Though the underlying mechanisms are not yet clear^2^. The resulting enhanced therapeutic ratio has been observed in mouse studies^3^. Studies in higher animals have shown improved normal tissue response^4^ and initial human studies have also demonstrated favorable response in both tumor control and normal tissue toxicity^5^.

In the context of *biodosimetry*, Improvised Nuclear Device (IND) exposures result in a broad range of dose rates^6^. As the main photon dose from a nuclear detonation is delivered in the form of prompt gamma rays, formed in the fraction of a microsecond before the fissile material is dispersed in the explosion^7^, it is critical to test assay performance using a realistic exposure time scale, rather than the common “1 Gy/min”.

In general, low dose rates result in increased repair of sub-lethal damage, with SSB and DSB repair occurring in a few minutes or hours respectively^8^, resulting in reduced DNA damage yields per unit dose. At high dose rates (~ Gy/s), some direct experiments^9^, and measurements of fast DSB repair times^10^ suggest that there will be increased effects from a dose delivered in ~ 1 s compared with the same dose delivered in ~ 1 min. Conversely, the FLASH effect would seem to indicate that at very high doses and dose rates, where oxygen depletion in the cellular environment plays a major role in radiation response, there may be a decline in DNA damage, formed in an oxygen-dependent processes. It thus becomes crucial to develop irradiation platforms that could deliver an LD50 dose, on microsecond time scales (modeling the blast) in addition to ones delivering dose over days (modeling fallout).

High dose rate is also extremely useful when modeling partial body exposures in mice, where the irradiation should be significantly faster than the circulation time of blood in the mouse (~ 15 s^11^). Otherwise, instead of irradiating a fraction ($$f)$$ of the blood to a given dose ($$D)$$, all the circulating blood is irradiated to dose $$D\times f$$—a completely different scenario.

Having recently developed irradiation platforms to study low dose rate effects^12,13^, we describe here a dedicated ultra-high dose rate (UHDR) irradiator, that we have implemented at the Radiological Research Accelerator Facility (RARAF). The irradiator is based on a decommissioned medical linear accelerator (linac), modified to operate in *FLASH* mode, using modifications similar to those developed at Stanford^14^ and Lund^15^ and a higher dose rate *SuperFLASH* mode using the approach of the Dartmouth group^15,16^. As opposed to the prior systems described in the literature, we are not using a clinically-active machine. We were thus able to make modifications incompatible with future patient use.

As described below, our UHDR irradiator allows irradiation of mice, cells, and blood vials at dose rates ranging from conventional (Gy/min) to > 100 Gy per few microsecond pulse.

As an example of the studies now open to us, we demonstrate reduced dicentric yields at ultra-high dose rates. A separate manuscript (Padilla, in process^17^) describes FLASH treatment of Glioma in mice.

## Methods

### Platform development

**Figure 1:**
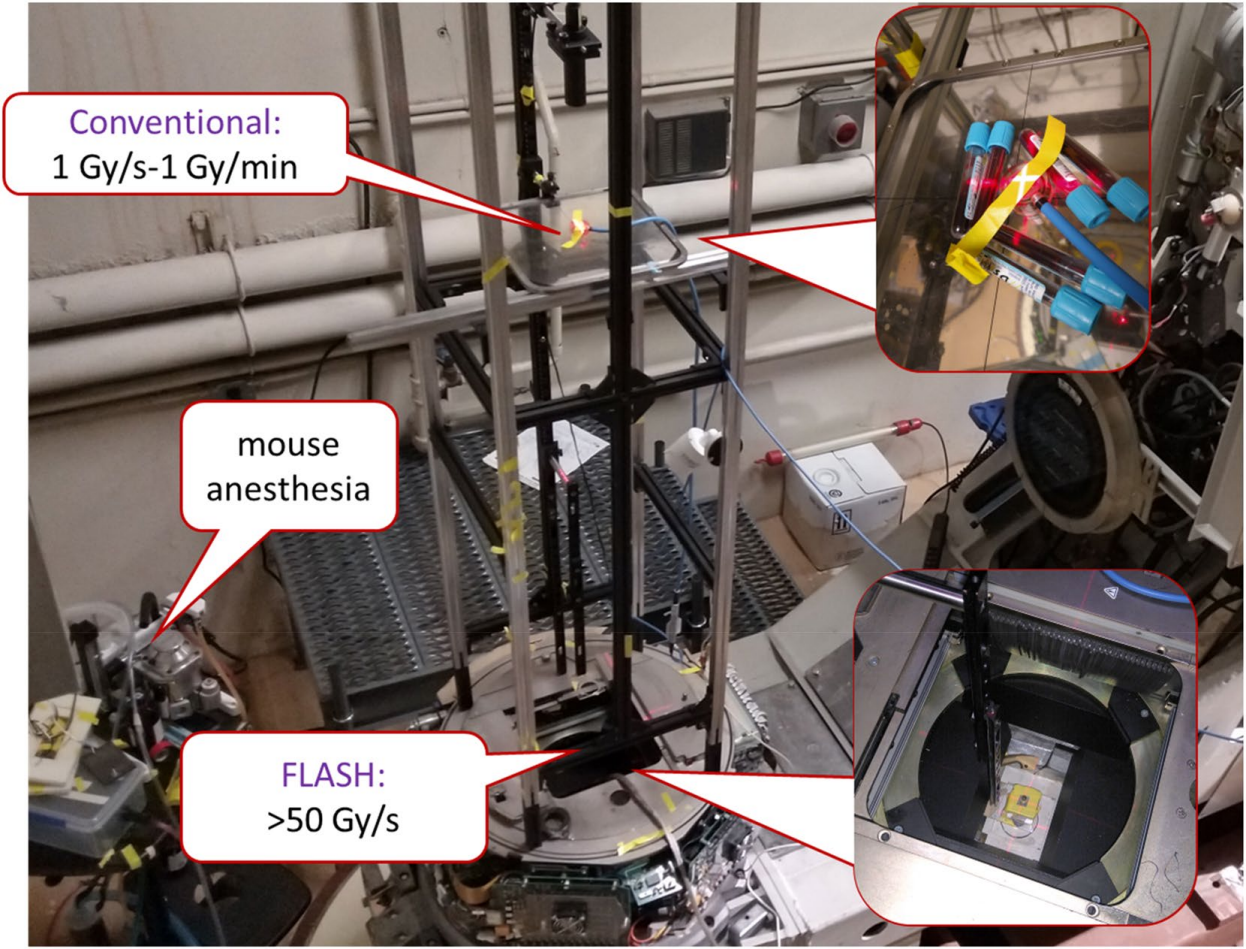
Photo of the ultra-high dose rate irradiator.

The RARAF UHDR irradiator is based on a retired Clinac 2100C (Varian Medical Systems, Palo Alto, CA, USA), recently installed in the accelerator hall at RARAF (Fig. 1). Due to the tight space, the bed was discarded, and the gantry rotation was disabled with the beam permanently pointing vertically up.

For most irradiations, the Clinac is operated in service mode, using the 9 MeV electron setting (*FLASH* mode), providing the best penetration into tissue below the 11 MeV photo-neutron threshold of ^56^Fe^18^. Higher dose rates, with a degraded dose depth profile are achievable when operating in 6 MV photon mode, with the target retracted (*SuperFLASH* mode). In this mode, the per-pulse electron current is increased by a factor of 50 and the repetition rate by an additional factor of two, nominally to overcome the low photon yield at 6 MeV, allowing ultra-high electron dose rates.

To minimize beam divergence, the foil assembly was removed from the carousel. The target piston was also decoupled from the Clinac control hardware and was set to be permanently in the “out” position. In this way the only material in the beam is the beam exit window and the integrated ionization chamber assembly.

**Figure 2:**
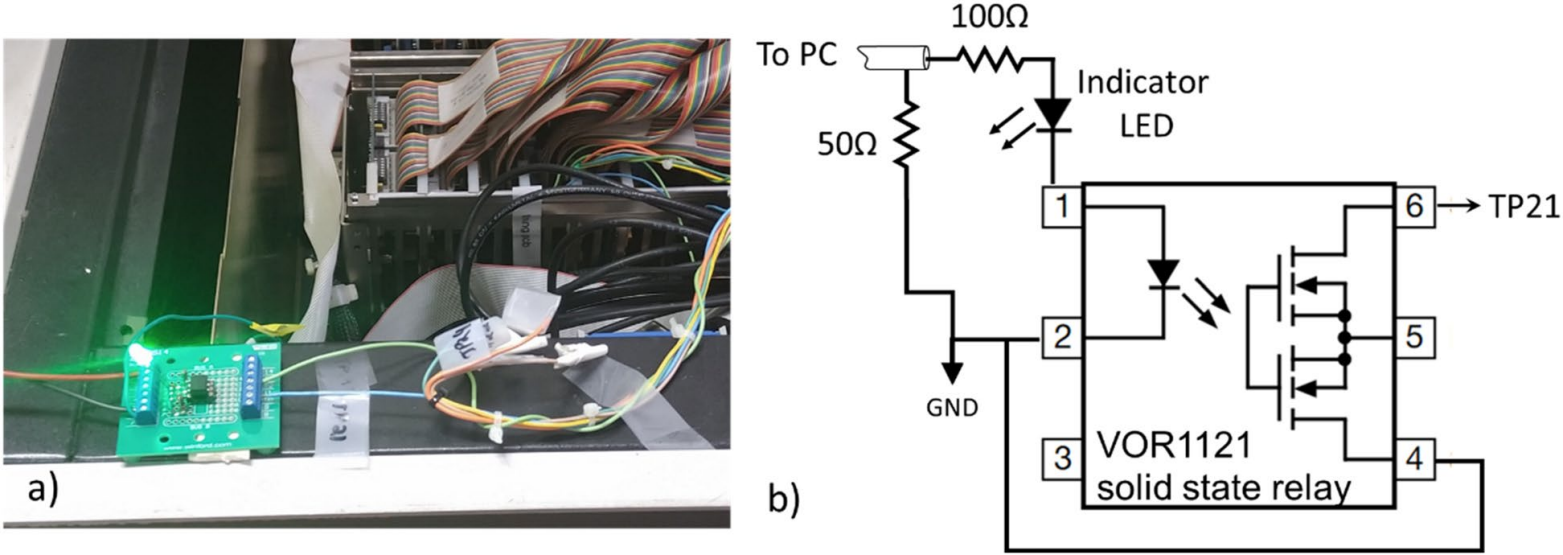
(**a**) Photo and (**b**) schematic of the interface circuit between the PC and the Clinac timing card. 5 V from the PC enables beam.

We have modified the timer interface card (Fig. 2), replacing the Varian generated GDLY CNT signal with our own signal. Under normal Clinac operation, this signal controls the delay between the electron gun firing and the klystron. When GDLY CNT is 0, the two are synchronized and radiation is delivered; when it is + 12 V the electron beam is generated out of phase with the klystron and no radiation is delivered.

**Figure 3:**
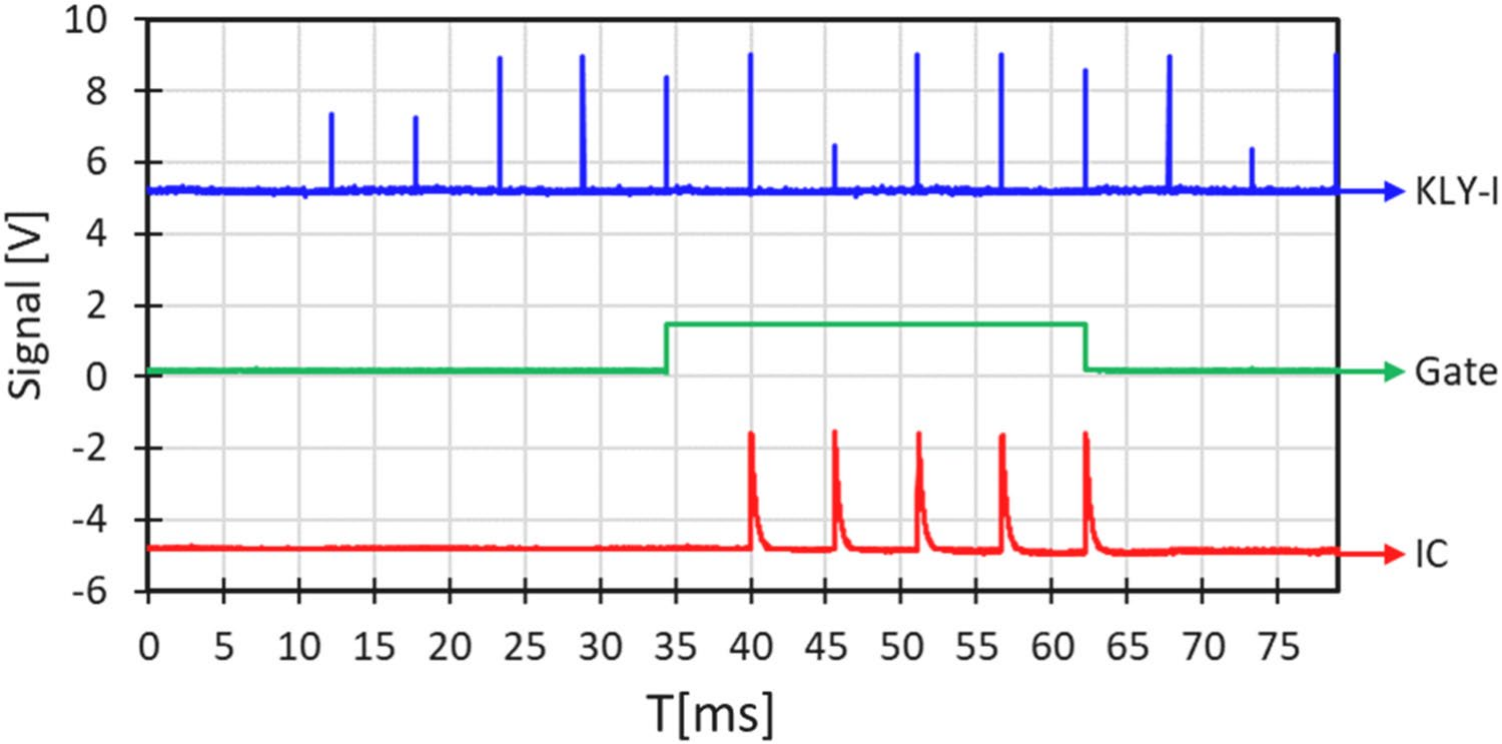
Oscilloscope trace of KLY-I, the CTR08-generated beam enable signal (Gate) and the built in ion chamber (IC). This irradiation was programmed to deliver 5 pulses after a delay of 5 pulses (25 ms). Variation in KLY-I pulse height is due to undersampling—pulses are all 5 V. KLY-I and IC traces offset by ± 5 V to facilitate viewing.

To generate the control pulse train (Fig. 3) we utilized a USB-CTR08 card (Measurement Computing, Norton, MA, USA). The card accepts as input the KLY I signal from the Clinac controller and turns the output on (+ 5 V) after a predetermined number of KLY I pulses for a predetermined number of pulses and then either stops or repeats. The output signal is used to actuate a solid-state relay (VOR1121A6; Mouser Electronics, Mansfield, TX, USA) which grounds TP21 on the timer interface card (beam on) or allows it to be pulled up to + 12 V (beam off).

### Dosimetry

**Figure 4:**
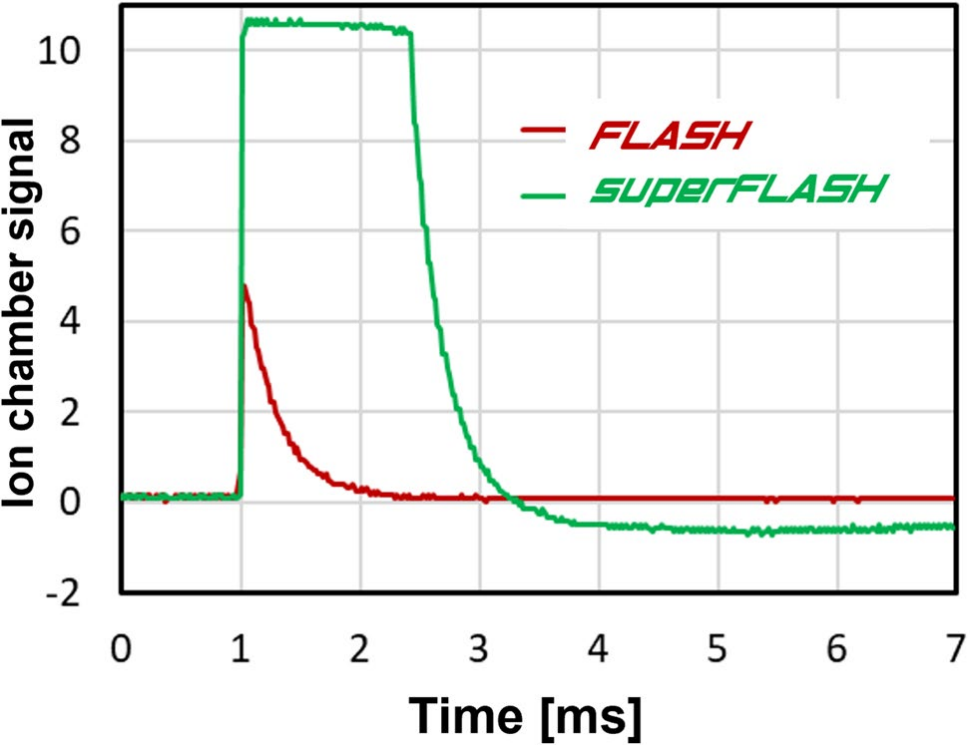
Signal from the built in ion chamber for the two operation modes.

In 9 MeV electron mode, beam intensity could be monitored using the built-in ion chamber. Specifically, test Point TP1 on the ion chamber control card was hooked up to a multichannel analyzer, and the average pulse height and number of pulses recorded for each irradiation. In *SuperFLASH* mode, the ion chamber is severely saturated and cannot be used (Fig. 4). An alternative beam monitor, as described by Yamada^19^ is under development.

NIST traceable dosimetry was performed, using a protocol based on AAPM TG-51^20^ using an Advanced Markus ion chamber (AMIC) and UNIDOS E electrometer (PTW, Freiburg, Germany). The ion chamber, operated at a polarization of − 300 V, was calibrated to absorbed dose in water, using a ^60^Co source at the MD Anderson Accredited Dosimetry Calibration Laboratory (ADCL). Measured dose was corrected for temperature, pressure, radiation quality (Table 1) and in some cases for recombination: at high dose per pulse, recombination corrections need to be taken into account when using an ion chamber to determine dose or dose rate. PTW states that the AMIC is certified to have ≥ 99% saturation (i.e. no recombination corrections need to be used) up to a dose rate of 5.56 mGy/pulse. TG-51 and TRS-398 recommend using the 2 voltage technique^20,21^, which assumes that the corrections due to recombination are small. At higher dose rates, more complex models^22–25^ can be used to calculate the required corrections. A better approach would be to engineer an ionization chamber in which recombination is smaller, typically by making it thin^26^.

**Table 1:** Beam quality correction for the AMIC.

Mode	Energy (MeV)	*R*_*50*_^†^ (cm)	${k}_{Co,e-}$^‡^
*SuperFLASH*	6	2.4	0.939
*FLASH*	9	3.9	0.922

^†^Interpolated from Fig. 9.

^‡^Interpolated from PTW paperwork.

**Figure 5:**
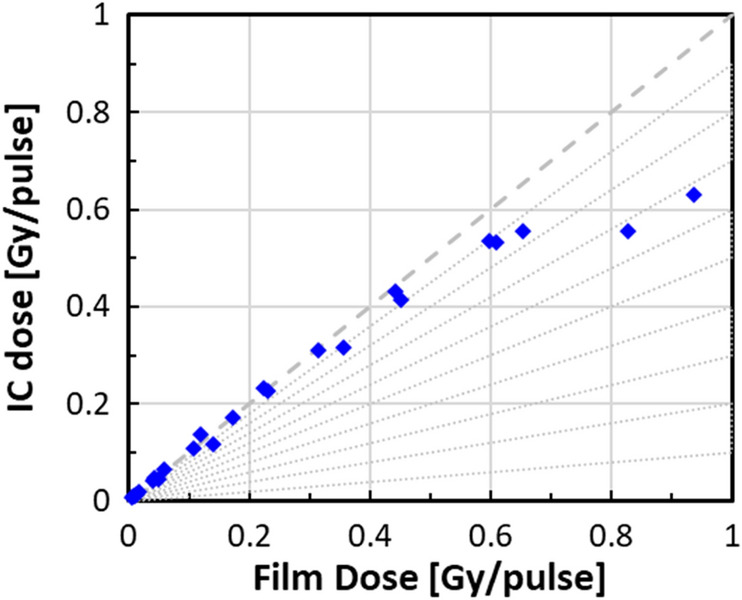
Measurement of recombination corrections to the AMIC. The Dashed line corresponds to 100% collection efficiency. The dotted lines represent 10% decreases in collection efficiency. Up to about 0.5 Gy/pulse the AMIC reports the correct dose.

We have followed the approach of Petersson^24^ and of DiMartino^27^ and measured the effects of recombination directly by irradiating EBT3 film and the AMIC simultaneously (Fig. 5). It was seen from these measurements that at dose rates smaller than about 0.4 Gy/pulse, no correction need be made. At a dose rate of 0.6 Gy/pulse the correction is ~ 10% (consistent with^24,27^) and at higher dose rates, the AMIC should not be used at all, both due to recombination and due to geometrical effects (in our setup dose rates above 0.5 Gy/pulse are obtained only with a spot size that is comparable in size with the AMIC sensitive region.

We therefore only use the AMIC for dose rates of up to about 0.5 Gy/pulse and for radiation fields > 3 cm in diameter (3 × sensitive volume diameter).

For dose rates above 0.5 Gy/pulse (90 Gy/s) and for smaller fields, we used EBT3 Radiochromic film (Ashland Specialty Chemicals, Wayne, NJ, USA) at doses below 20 Gy and OC-1 OrthoChromic film (Orthochrome Inc., Hillsborough, NJ, USA) at high doses. We selected OC-1 over the more common EBT3-XD, due to its higher saturation dose and better high dose rate performance^28^.

Each batch of film was calibrated by irradiating film, within a solid water phantom and the AMIC at a dose rate of approximately 1–5 Gy/min (SSD = 171 cm), using a large field size (typical calibration curves are shown in Fig. S1).

Film was scanned using an Epson V700 scanner in transmission mode (EBT-3) or reflection mode (OC-1). The scanner was operated several times to warm up the lamp and then films (including an unirradiated control film) were placed on the scanner bed (EBT-3 under a sheet of glass) and scanned at 300 DPI and a bit depth of 48 bit. Only the red channel was used for dose reconstruction. Optical density was taken as negative log 10 of the ratio of red color value in the irradiated and unirradiated films. Dose vs. optical density was calibrated using a first order rational function $D=\frac{a-b\times OD}{OD-c}$. A matlab script was then used to convert scanned film images to dose maps and to generate dose histograms in selected regions of the image.

### Safety

Our accelerator hall was originally built of interlocking 70 cm thick reinforced concrete shield blocks. As this is not strictly sufficient shielding in conventional clinical operations^29^, we have mapped the leakage radiation and routinely lock off high radiation rooms (specifically the room directly above the Clinac head) during operation. Lower radiation areas are cordoned off as “no linger” zones. Similar to clinical installations, the DOOR interlock is used to ensure that the accelerator hall is vacated and locked during use. A Geiger-Muller based area monitor (Luldlum Measurements Inc, Sweetwater, TX, USA) is located adjacent to the Clinac with a lighted sign visible from outside the accelerator hall. Additional monitors are placed in the rooms adjacent to the vault, including at the console.

As we are only irradiating using electrons and have seen excessive stray radiation outside the accelerator hall only when generating photon beams, we have disabled the target piston, such that the target is always in the out position.

### Sample positioning

**Figure 6:**
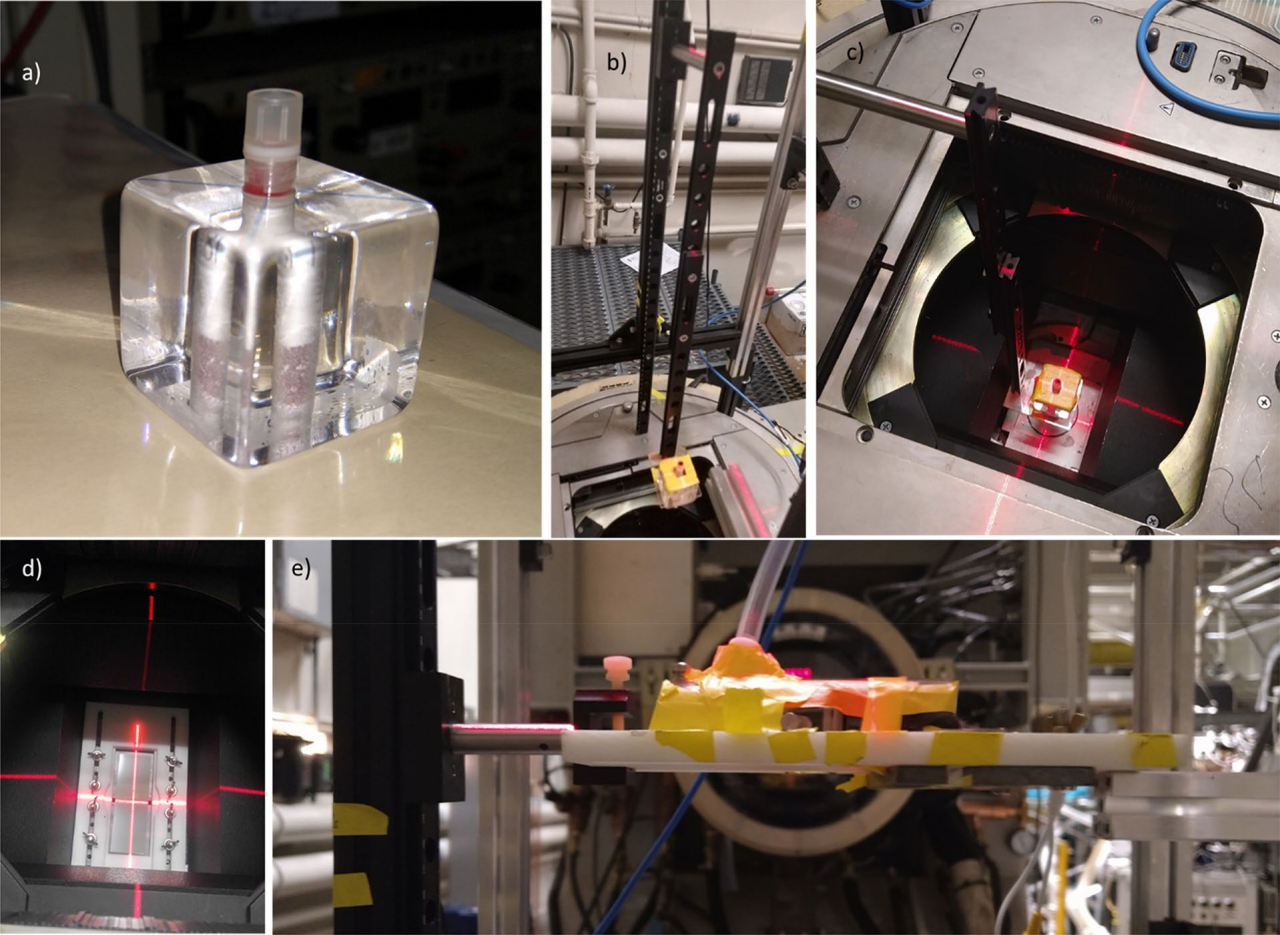
(**a**) Holder for matrix tube at an SSD of (**b**) 75 cm and (**c**) 20 cm. (**d**) shielded holder for mice. (**e**) shielded holder with anesthetized mouse being irradiated hemi-body at 10 Gy/s (SSD = 90 cm).

To allow for reproducible sample positioning both inside and outside the Clinac head, we have constructed a scaffold, using 25 mm extruded aluminum optical construction rails (Thorlabs, Newton, NJ, USA). A graduated optical dovetail rail (Thorlabs) was bolted on the construction rail with the sample holder mounted on a dovetail rail carrier (Fig. 6b).

We report sample positions in terms of Source to Surface Distance (SSD) with SSD = 100 cm at isocenter, which Varian specifies to be at the rotation axis of the gantry.

Test tubes were placed in a 38 mm bored acrylic cube (McMaster-Carr, Chicago, IL, USA; Fig. 6a/c). In addition, a custom holder was designed, and 3D printed (Protolabs Inc, Maple Plains, MN, USA) using PA12 Nylon (Fig. 6d). This holder allowed irradiation of larger objects, Petri dishes or a mouse anesthesia jig (Precision X ray Irradiation Inc, North Branford, CT, USA; Fig. 6e). Adjustable 6.4 mm thick lead screens were added for beam collimation.

To facilitate sample alignment to the beam we have mounted a 5mW red cross projection laser (Laserglow Technologies, North York, ON, USA) to the top of the scaffold. The laser was aligned to the beam using in situ irradiated film.

### Dicentric analysis

This study was approved by Columbia University’s Institutional Review Board (IRB) protocol IRB-AAAR0643. All methods were performed in accordance with the relevant guidelines and regulations. Peripheral blood was obtained after informed consent from two healthy volunteers (male, 32 year and female, 54 year) with no recent history of exposure to ionizing radiation or clastogenic agents. Freshly-drawn blood was heparinized (vacutainers with sodium heparin anticoagulant (Becton Dickinson, NJ, USA)), aliquoted into Matrix Storage Tubes (Fisher), and exposed to 3 Gy or 8 Gy 9-MeV electrons at a wide range of the dose-rates. SSD and # of pulses for each dose rate are given in Table S1). The control samples were sham-irradiated and received 0 Gy dose.

After irradiation, the blood (0.5 mL) was stimulated with a mitogen (phytohemagglutinin, PB-MAX karyotyping medium) and incubated at 37 °C, 5% CO^2^ for 48 h. 3 h before fixation, cells were arrested in metaphase with 0.1 mg/mL colcemid (Gibco). Following the treatment, cells were swollen for 20 min in hypotonic solution (0.075 M KCl) and fixed with methanol:acetic acid (3:1). 20 μL of fixed cells were dropped onto glass slides, dried at an ambient temperature of 25 °C and relative humidity of 55%. Dried slides were then fixed in 4% formaldehyde and dehydrated in ethanol (70–85–100% for 2 min each). Prepared chromosome spreads were stained with a PNA probe hybridization cocktail (FITC-labelled centromere and Cy3-labelled telomere probes (PNABio) in buffer), covered by a cover glass, denatured for 3 min at 80 °C, and left at a room temperature in the dark for 2 h. After that, the slides were washed twice in 70% formamide, then twice in TBST (with 0.05% Tween™-20), and counterstained with Vectashield^®^ Antifade Mounting Medium with DAPI (4′,6-diamidino-2-phenylindole).

Images were acquired on a Metafer 4 Scanning System (MetaSystems) equipped with a Carl Zeiss Axioplan Imager Z1, a CoolCube 1 Digital High-Resolution CCD Camera, and a Zeiss Plan-Apochromat 63 × oil immersion objective. Metaphases were located using an automated classifier comprised of two acquisition modules, MSearch and AutoCapt. Captured images were analyzed using Isis software (MetaSystems). Metaphase cells were selected at low magnification (10×) and examined for quality under higher magnification (63×) to exclude cells with overlapped chromosomes or not clearly distinguishable chromatids. Only complete metaphases (46 centromere spots) were used for analysis. The samples were blinded to the scorer and decoded afterward. For scoring, 50 cells per donor were analyzed (QuickScan method^30^) using the morphological criteria specified by IAEA cytogenetic dosimetry manual^31^. The dicentric yields were subsequently adjusted by conversion of multicentric aberrations into the dicentric equivalent (tricentric chromosome is equal to two dicentrics, tetracentric chromosome is equal to three dicentrics, etc.). The yields of dicentrics and their distribution among cells have been used to calculate the dispersion index (σ^2^/y) and the normalized unit of this index (u) using the equation recommended by IAEA^31^.

For dose–response curves, the yields of dicentrics (Y) was used to calculate the coefficients of the linear-quadratic mathematical function ($Y = \alpha D + \beta {D}^{2}$, where *D* is a dose and *α* and *β* are the linear and quadratic coefficients, respectively) in CABAS v.2 software^32^. The uncertainties for $\alpha /\beta$ quotients were estimated by propagating the relative errors in quadrature: ${\sigma }_{\alpha /\beta }=\frac{\alpha }{\beta }\sqrt{{\left(\frac{{\sigma }_{\alpha }}{\alpha }\right)}^{2}+{\left(\frac{{\sigma }_{\beta }}{\beta }\right)}^{2}}$, where $\sigma$ represents the standard error.

## Results

### Reproducibility

**Figure 7:**
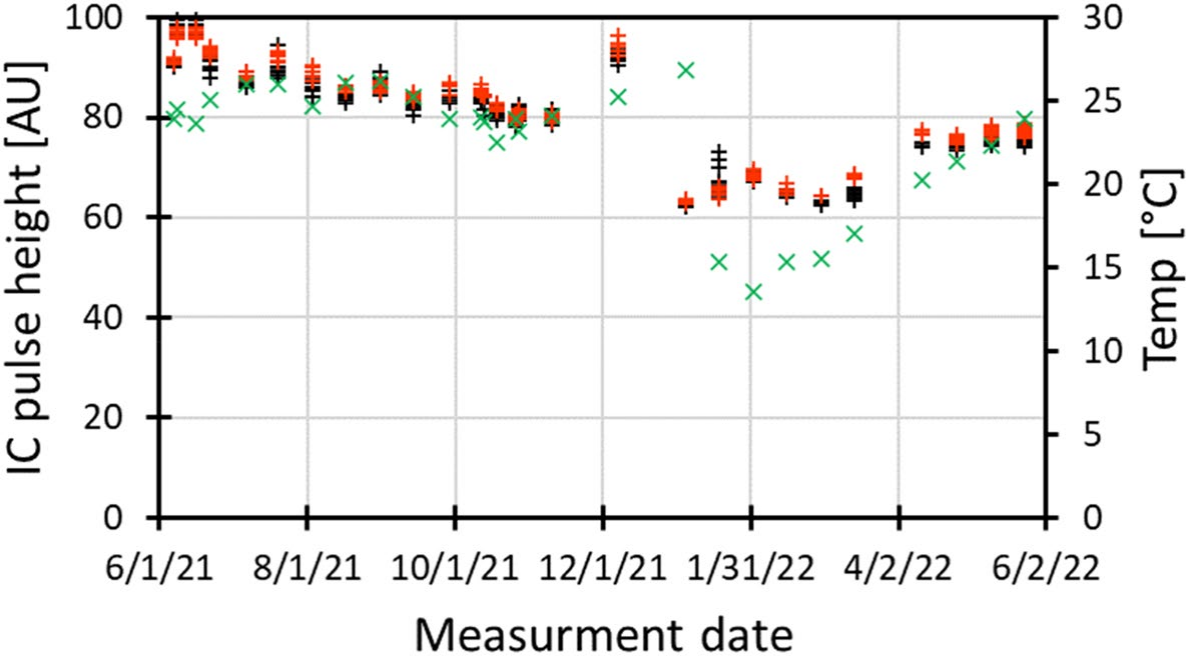
Average pulse height from the built in ion chamber for 2 pulse irradiations (red) and 3 pulse irradiations (black) in FLASH mode, over 12 months of experiments.The Xs denote the temperature in the room at the beginning of the experiment.

Figure 7 shows the average pulse height measured in *FLASH* mode, using the integrated ion chamber over a set of 26 experiments conducted in the 2nd half of 2021 and 1st half of 2022. In each experiment, 4–10 samples were irradiated using 2 pulses and 4–10 using 3 pulses. The graph shows fluctuations of 1–2% (relative standard deviation) in pulse height within a set of irradiations and a slow drift over several months that appears correlated with room temperature.

In *SuperFLASH* mode, the built-in IC always reads the same, saturated, pulse height (Fig. 4), so reproducibility can only be tested using film. Based on a smaller set of film measurements, pulse height variations were roughly ± 15% within a single run.

### Beam characterization

#### Pulse timing

**Figure 8:**
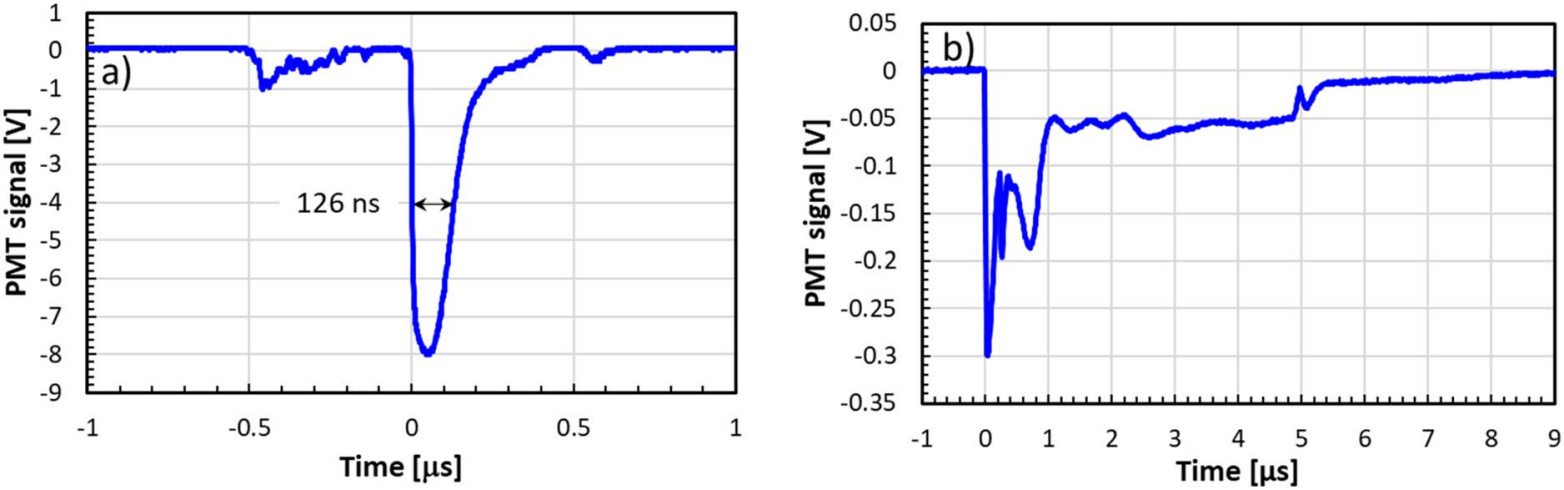
Oscilloscope trace of a single Clinac pulse measured directly using a scintillator and PMT tube. (**a**) FLASH mode, (**b**) SuperFLASH mode.

Figure 4 (above) shows an oscilloscope trace of the built-in IC signal for 1 pulse in *FLASH* and *SuperFLASH* modes. The pulse width is dominated by the ion chamber shaping electronics and cannot be used to estimate the actual pulse duration. Using a nanosecond timing scintillator (EJ-301; ELJEN Technology, Sweetwater, TX, USA) and photomultiplier tube (RCA 8575), we were able to measure pulse width as 0.13 µs FWHM (Fig. 8a) in *FLASH* mode. In *SuperFLASH* mode we have seen saturation of the PMT resulting in a significantly broadened pulse. Reducing the PMT gain significantly and placing it at a SSD of 171 cm we were able to measure pulse timing. We have consistently seen that in this mode ~ 40% of the dose is delivered in 1 µs with the rest delivered over an additional 4 µs.

#### Percent depth dose

**Figure 9:**
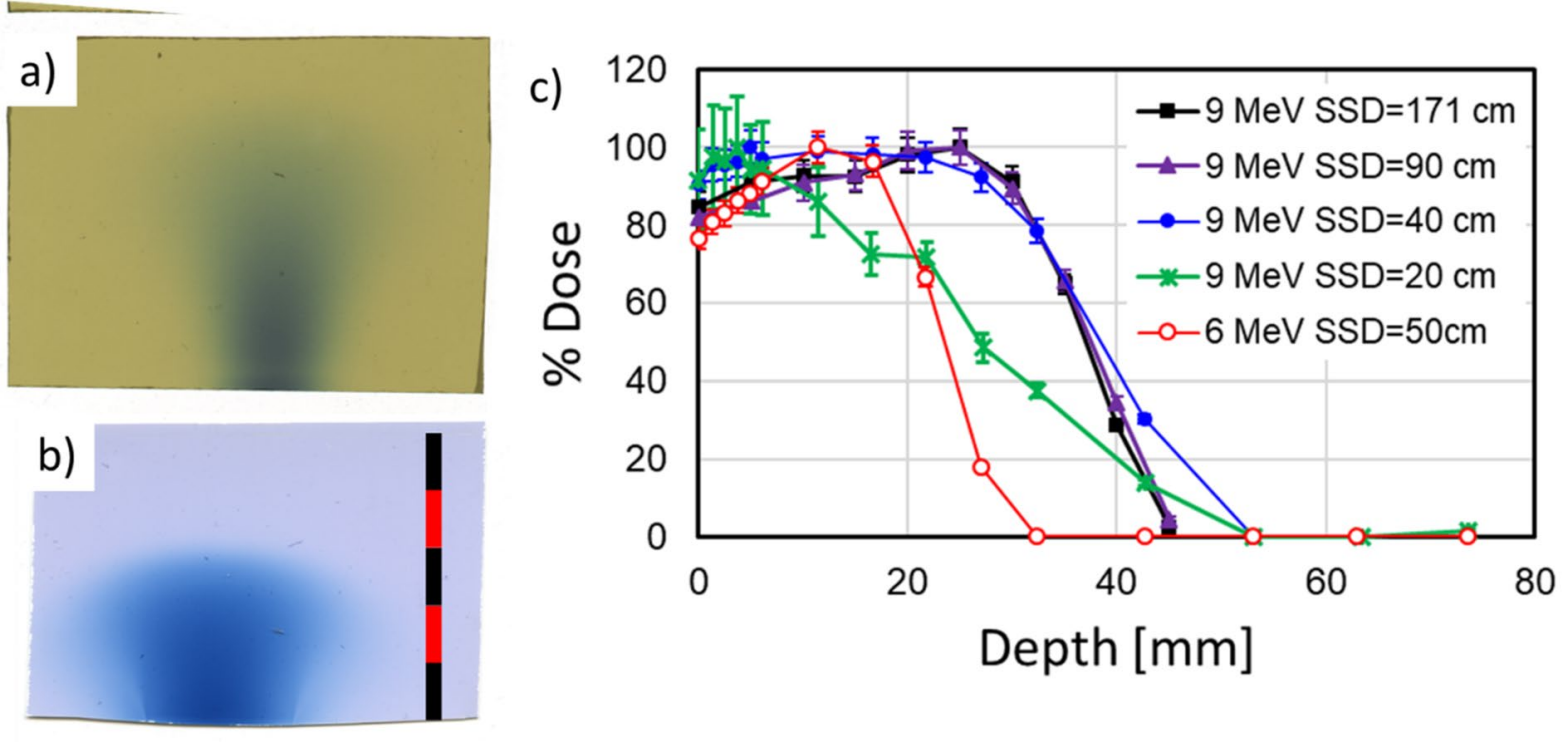
Percent depth dose measurement. (**a**)EBT3 and (**b**) OC1 film irradiated parallel to 9 and 6 MeV beams, respectively. Scale bar is 5 × 1 cm. (**c**) dose evaluated from EBT3 film irradiated perpendicular to the beam within a water equivalent polystyrene phantom. 6 MeV: red—SSD = 50 cm; 1 pulse; peak dose 7.7 Gy; 9 MeV beams: green—SSD = 20 cm, 3 pulses, peak dose: 9 Gy; blue—SSD = 40 cm, 30 pulses, peak dose 8.5 Gy; purple—SSD = 90 cm, 200 pulses, peak dose 8 Gy; black—SSD = 171 cm 926 pulses, peak dose 7.6 Gy.

PDD was visualized using radiochromic film sandwiched between sheets of water equivalent polystyrene (RW3, LAP laser, Boynton Beach, FL, USA). The film was placed in the plane of the beam, at an SSD of 20 cm and irradiated with a single pulse of 6 or 9 MeV electrons (Fig. 9a,b). At this position, the beam diameter is about 2 cm. It is also obvious from Fig. 9a,b that the 6 MeV beam scatters significantly more than the 9 MeV beam.

PDD measurements were then performed by irradiating multiple sheets of EBT3 film within a stack of 1 mm, 5 mm and 10 mm sheets of RW3, perpendicular to the beam (Fig. 9c). R_50_ was measured as 39 mm for 9 MeV (SSD = 40 cm) and 24 mm for 6 MeV (SSD = 50 cm). At SSD = 20 cm, R_50_ is significantly lower (27 mm for 9 MeV), due to the divergence of the beam.

#### Dose rate vs spot size

**Figure 10:**
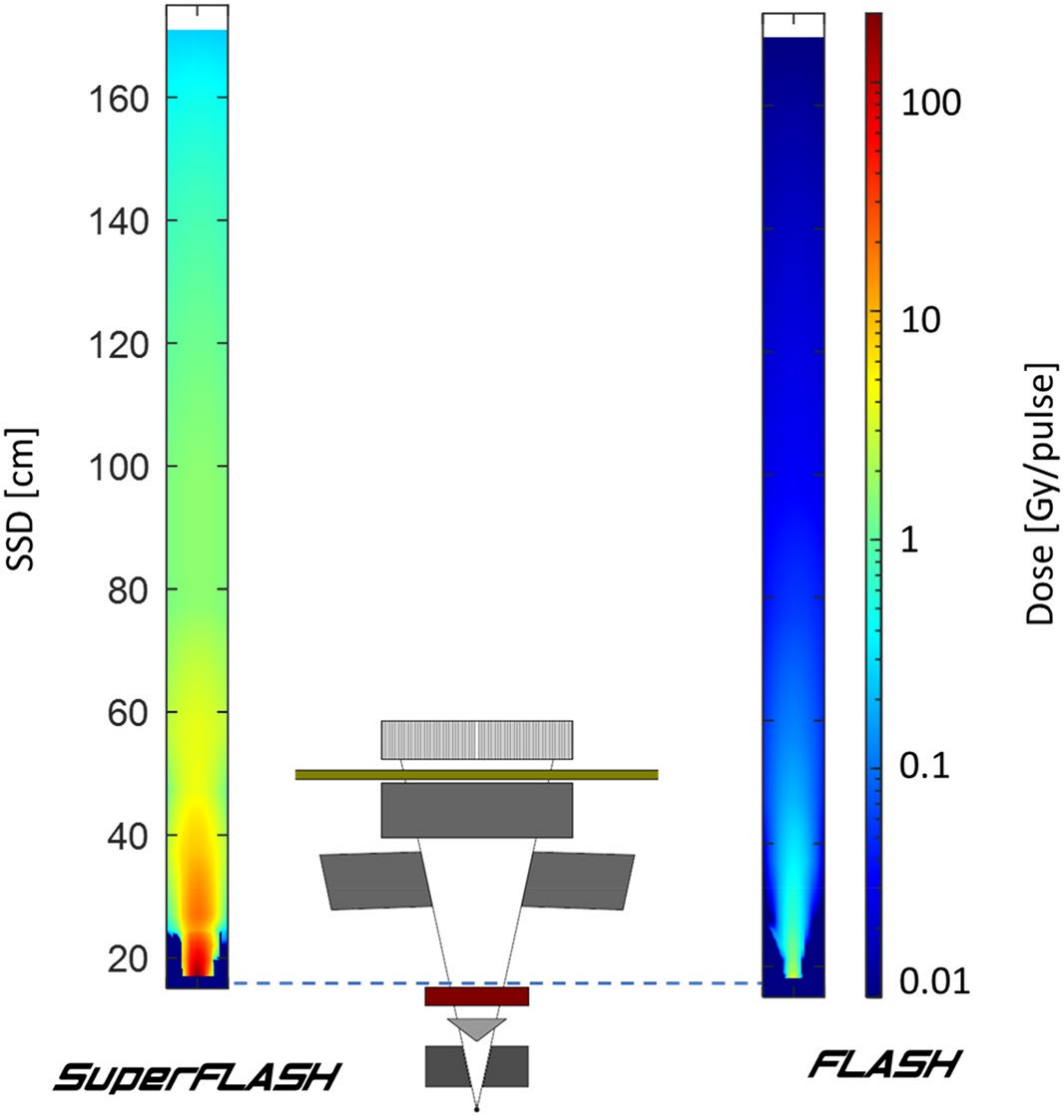
Heat map of dose rate in the two operation modes, adapted from^33^, with permission from Elsevier.

Figure 10 and Fig. S2 show 6 and 9 MeV electron beam brightness at various positions in the system, with the isocenter at 100 cm. Distributions across the beam at different SSD values are shown in Fig. S2.

### Dicentric analysis

**Figure 11:**
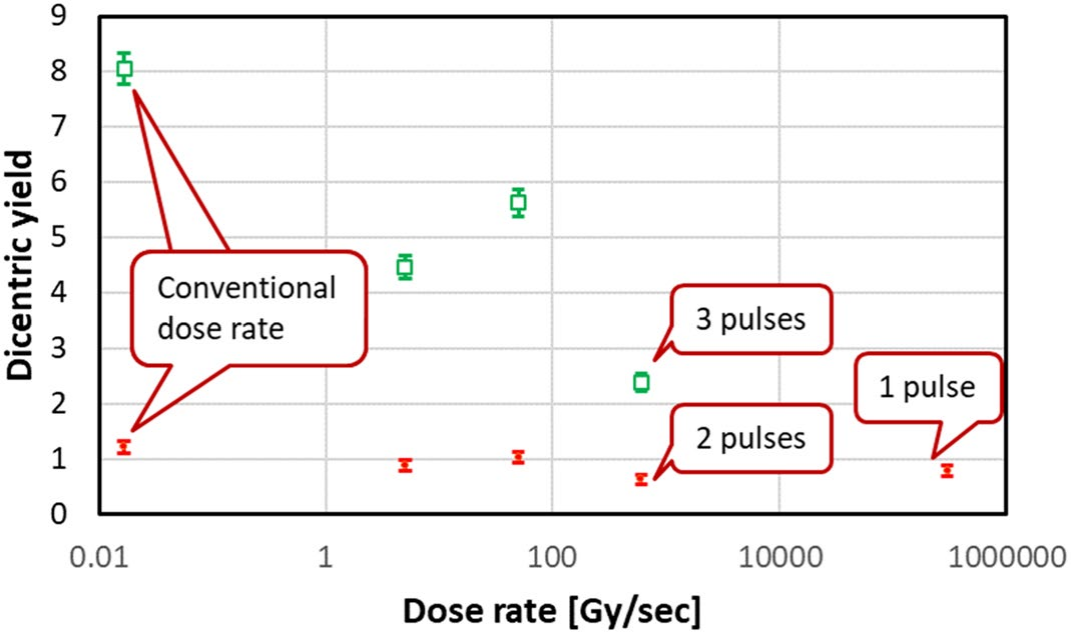
Dicentric yields for 3 Gy (red closed symbols) and 8 Gy(green open symbols) irradiations at different dose rates. No dicentrics or multicentrics (yield = 0 ± 0) seen in control samples.

The numbers and distribution of dicentrics among cells are given in Fig. 11 and Table S1. The resulting dose–response curves are shown in Fig. S3. The α and β coefficients, and the α/β quotients for each dose rate are given in Table 2.

**Table 2:** Values of the coefficients α and β in the equation Y = αD + βD^2^ and α/β quotients which gives the dose–response curves for each dose rate.

Dose rate	SSD (cm)	α ± SE × 10	β ± SE × 10	α/β* ± SE
1 Gy/min	171	0.47 ± 0.63	1.20 ± 0.10	0.4 ± 0.5
5 Gy/s	90	1.35 ± 0.52	0.53 ± 0.08	2.6 ± 1.1
50 Gy/s	40	1.28 ± 0.57	0.72 ± 0.09	1.8 ± 0.8
“600 Gy/s”^†^	20–24	1.57 ± 0.44	0.18 ± 0.07	8.7 ± 4.2

*α/β quotient is the dose at which contributions from single-track (α coefficient) and double-track (β coefficient) events are equal.

^†^600 Gy/s is a rough approximation of the average dose rate. Dose was delivered as either two 1.5 Gy pulses or three 2.7 Gy pulses at a pulse duration of 130 ns and repetition rate of 180 Hz.

## Discussion

### Operation of the UHDR irradiator

To date, the UHDR irradiator at RARAF has been operational for over 2 years. After initial optimization of the beam settings, we have been able to operate with minimal adjustments. As seen from Fig. 7, reproducibility of the beam intensity is quite good. The initial drop in June 2021 is suspected to be related to burn-in of the high voltage diodes in the DeQing circuit driving the klystron, which were replaced in the spring of 2021. The slow variation appears correlated with the room temperature in the accelerator hall. This thermal effect seems to bottom out at 20 °C, which is the minimal allowed temperature for the cooling water.

This variation is not due to air density variations in the built-in ion chamber as (a) those are much smaller than the variation in beam output and (b) we have routinely seen good correlation between the built-in ion chamber signal and dose measured using film or the AMIC [the latter up to a dose rate of 0.5 Gy/pulse (90 Gy/s)]. Rather, it may be attributable to thermal effects in the electron gun or acceleration structure.

### Optimization of beam delivery

For each experiment, we optimized the sample position, pulse structure, and delivered number of pulses, while monitoring both the AMIC and the pulse height spectrum from the built-in ion chamber. In general, we have seen a good correlation between the delivered dose, at a given position, and the integral of the pulse height spectrum.

When operating in the 9 MeV electron mode, we have seen that the optimal beam brightness is obtained when allowing the electron gun to run for 20 s (3600 pulses) prior to generating the beam. This likely allows the Automated Frequency Control (AFC) to stabilize, as evidenced by the observed reduction in the AFC DIFF readout. It is likely that this can be better controlled by manual tuning of the frequency.

Anecdotally, when delivering short (< 1 s) bursts, reproducibility in beam intensity seems to be better if there is a 2–3-min gap between irradiations, we suspect that this is due to thermal effects in the electron gun, as evidenced by the fact that longer (> 1 s) bursts display a reduction in beam intensity with time.

#### Conventional dose rate

For irradiation at conventional dose rates (1–2 Gy/min), samples were placed at an SSD of 171 cm. At this position, the nominal dose rate is about 6 mGy/pulse and the field size is large, so that several test tubes, a flask or a multiwell plate could be placed alongside the AMIC. The electrometer was operated at medium scale and the pulse repetition rate was adjusted to 50–100 pulses off for every on pulse (repetition rate 2–4 Hz), such that the AMIC was reading the required dose rate. Repetition rate was adjusted periodically to compensate for drift in the delivered dose per pulse. Beam was stopped manually when the desired dose was achieved, typically with a precision better than 0.1 Gy.

#### Moderately high dose rate

For dose rates of approximately 1 Gy/s, human reflexes (and the refresh rate of the UNIDOSE E) are not fast enough to allow stopping the beam manually and there is sufficient drift in the beam intensity that delivering a known number of pulses does not necessarily result in the exact required dose.

To deliver a predetermined dose at 1 Gy/s we utilized the DOS1 interlock. Samples were irradiated at a pulse repetition rate of 180 Hz (the maximal repetition rate for 9 MeV electrons), in the same geometry as the conventional dose rate samples (SSD = 171 cm). We first irradiated the AMIC alone to determine the correct Monitor Unit (MU) count for the prescribed dose (25–30 MU/Gy with small day-to-day variations). We then irradiated the sample and AMIC side by side with our software set to deliver an excess of pulses and the DOS1 interlock set to trip at the required MU count. This typically resulted in a few % accuracy in delivered dose.

#### High dose rate

Dose rates of 20–100 Gy/s could be delivered with the sample placed inside the Clinac head (SSD = 40–80 cm) roughly in the position of the MLC or Y jaw). The field size in this case is too small for irradiating the sample and the AMIC simultaneously. Prior to each set of irradiations, the AMIC was used to find the position within the head and the required number of pulses for the prescribed dose rate and dose. As noted above, and as seen by Petersson^24^, this is at the upper end of usability of the AMIC (0.5 Gy/pulse or 90 Gy/s).

Typically, beam was delivered by waiting 20 s with the klystron and electron gun on but out of synchronization and then delivering the required number of pulses. This resulted in a maximal average pulse height from the built-in ion chamber, although pulse height was seen to start drifting after about a second of beam-on time.

#### Ultra high dose rates

With the sample at or below the X jaw (SSD = 20–30 cm), average dose rates of 200–600 Gy/s (1–3 Gy/pulse, 5.6 ms between pulses) could be obtained using 9 MeV electrons. In this case, dosimetry cannot be done using the AMIC at all, as the beam size is comparable to the AMIC sensitive volume and dose rate is much higher than 0.5 Gy/pulse, where collection efficiency plummets^24^. We, therefore, determined dose rate by irradiating EBT3 film in a solid water phantom. As above, beam was delivered using a 20 s delay preceding the radiation pulses.

#### SuperFLASH mode

Even higher dose rates could be achieved when operating the Clinac in 6 MeV photon mode, with the target removed. In this case the beam repetition rate is 360 Hz and the dose per pulse is also significantly higher (up to 150 Gy/pulse at SSD = 20 cm). Dosimetry was similarly performed using OC-1 film, which has better high dose performance, as compared to EBT3 and EBT3-XT^28^.

#### Dark current

As noted above, in most irradiations, the Clinac is operated with the electron gun and acceleration asynchronous for most of the irradiation time. During this “off time” we have seen negligible dose rate (< 10 mGy/min) on the AMIC, even when placed deep inside the head (SSD = 20 cm). This is because even if electrons get some acceleration, they cannot be sufficiently accelerated to match the magnetic rigidity required to pass through the 270° bending magnet in the Clinac head.

A secondary concern may be activation of the Clinac head, induced by high dose and dose rate irradiations. Indeed, the area monitor adjacent to the Clinac routinely reads a low-level activation (< 1 µSv/h) for a few minutes following some irradiations. This is monitored but does not affect irradiations.

### Selection of dose rate

**Figure 12:**
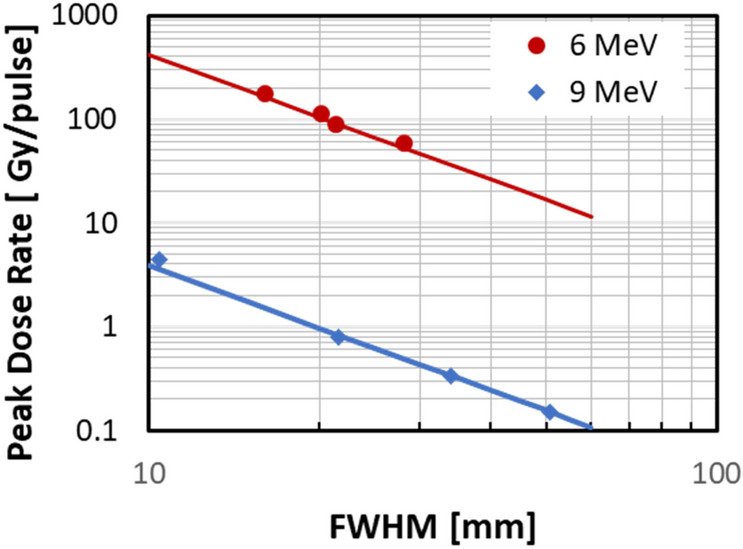
Peak dose rate vs Full Width at Half Max (FWHM) in FLASH and SuperFLASH modes. Reprinted from^33^ with permission from Elsevier.

The electron beam from the Clinac has a gaussian profile (Fig. S2) with the beam area increasing quadratically with SSD. Figure 12 shows the inverse square relation between dose rate and beam diameter. When designing an experiment, our goal is to obtain the maximal dose rate while maintaining <  ± 10% variation in beam intensity across the sample. This limits the maximal dose rate (in FLASH mode) depending on target size. Thus, a matrix tube (~ 1 cm diameter) can be irradiated to a maximal dose of 3 Gy/pulse at SSD = 20 cm. Larger objects like 35 mm Petri dishes or T12.5 flasks are limited to doses of about 0.25 Gy/pulse (SSD = 50 cm) and so on. In particular, mouse TBI is only available at dose rates of up to about 10 Gy/s (SSD = 90 cm) using 9 MeV electrons.

It should be noted that average dose rate is only a meaningful quantity if the number of pulses is large. When delivering dose in a few pulses, the full pulse structure (repetition rate, dose per pulse and pulse duration) needs to be reported. In this paper we use quotation marks to denote average dose rate when it is an inappropriate quantity but still useful as a designator, in this case we define the average dose rate as the dose delivered divided by the time between the first and last pulses.

### Depth-dose

One potential problem with electron-based UHDR irradiations is the shallow penetration of electron beams, compared to photon beams. As seen in Fig. 9 and Table 1, the penetration of electron beams available to us are only a few cm. This is particularly problematic with the *SuperFLASH* beam which only penetrates about 2.5 cm in tissue. For murine tumor irradiations, this is generally not a problem as the mouse can always be oriented such that the tumor is within the beam. Similarly, for irradiation of liquid samples (blood or cell cultures), proper selection of the aliquot size and vial shape can ensure that the entire sample is being irradiated to a uniform dose. When total-body-irradiating mice, however, the *SuperFLASH* mode may induce a large variation in dose in the mouse. For mouse TBI irradiations we are, therefore, testing alternative mouse holders where the mouse is splayed and slightly thinner than the conventional mouse holder (Fig. 6e). Initial tests have shown that at least with younger mice (7 week) the entrance and exit doses are within 10% of each other.

### Dose uncertainties

The main uncertainty we have encountered in our dosimetry is due to the beam geometry. At small SSDs, required for ultra-high dose rates, the beam diameter is small. For example, at an SSD of 20 cm the beam diameter (FWHM) is about 1 cm. This means that when irradiating samples in a vacutainer (10 mm ID) there is a ± 14% uncertainty in dose (measured as the standard deviation within a 1 cm circle on the film). Use of a matrix tube (6 mm ID) reduces this to ± 8% (Table 3). Irradiating at SSD = 20 cm also raises the risk for sample misalignment within the beam, with a 1 mm misalignment corresponding to 2% change in the average dose and a 2 mm misalignment resulting in a 6% change in dose.

**Table 3:** Uncertainty budget for dicentric study.

Source of error	Matrix tube SSD = 20 cm (%)	Matrix tube SSD = > 40 cm (%)
Lateral dose variation	8	< 4
Longitudinal dose variation	11	< 3
Misalignment	2	Negligible
Beam repeatability	2	2
Dosimetry calibration	4	4
Total	14	< 7

At SSD = 20 cm there is also a significant change in dose along the beam, due to its divergent nature. This can be seen when comparing the green and blue curves in Fig. 9c. This longitudinal variation would contribute an uncertainty of 11% to a 2 cm thick sample. At larger SSD values, this becomes less of a problem – both due to the larger FWHM of the beam and due to the slower decline in dose rate with SSD. For an SSD of 40 cm, the lateral variation in dose for a 1 cm wide object is ± 4% and a longitudinal variation for a 2 cm thick object is 3%.

These dose uncertainties are inherent to this method of generating high dose rate beams and can only be overcome by using a custom-built accelerator, designed for generating parallel high flux beams, such as the one at Lausanne^34^.

In *SuperFLASH* mode, there is currently an additional variation due to repeatability of the beam of about ± 15%. This is likely due to a combination of thermal effects and improper stabilization of the AFC. While we work to resolve this issue, a temporary workaround is to irradiate excess samples, with film included in each irradiation and to select those samples that received the correct dose post irradiation.

### Dicentric yields

Overall, the detailed aberration analyses revealed a marked decrease in dicentric yields at higher dose rates (p values < 0.05, estimated using T-test assuming equal (independent) variances). This is consistent with the so-called FLASH effect seen in many biological endpoints^1,2^.

The data in Table S1 on dicentric aberration yields for the different radiation doses and dose rates were fitted to the linear-quadratic model. The dicentric aberrations were distributed randomly among the cells according to Poisson statistics and changed with the dose. At a lower dose (3 Gy), throughout all dose rates, a maximum amount of dicentrics per cell was consistent (up to 3–4 dicentrics/cell). The aberration distribution patterns were predominantly similar; however, at higher dose rates, the aberration yields were slightly lower than at conventional 1 Gy/min samples. With the exception of a single cell with tricentric in a 1 Gy/min sample, the aberrations, formed at different dose rates at 3 Gy, were solely dicentrics, and multicentric chromosomes were not observed.

**Figure 13:**
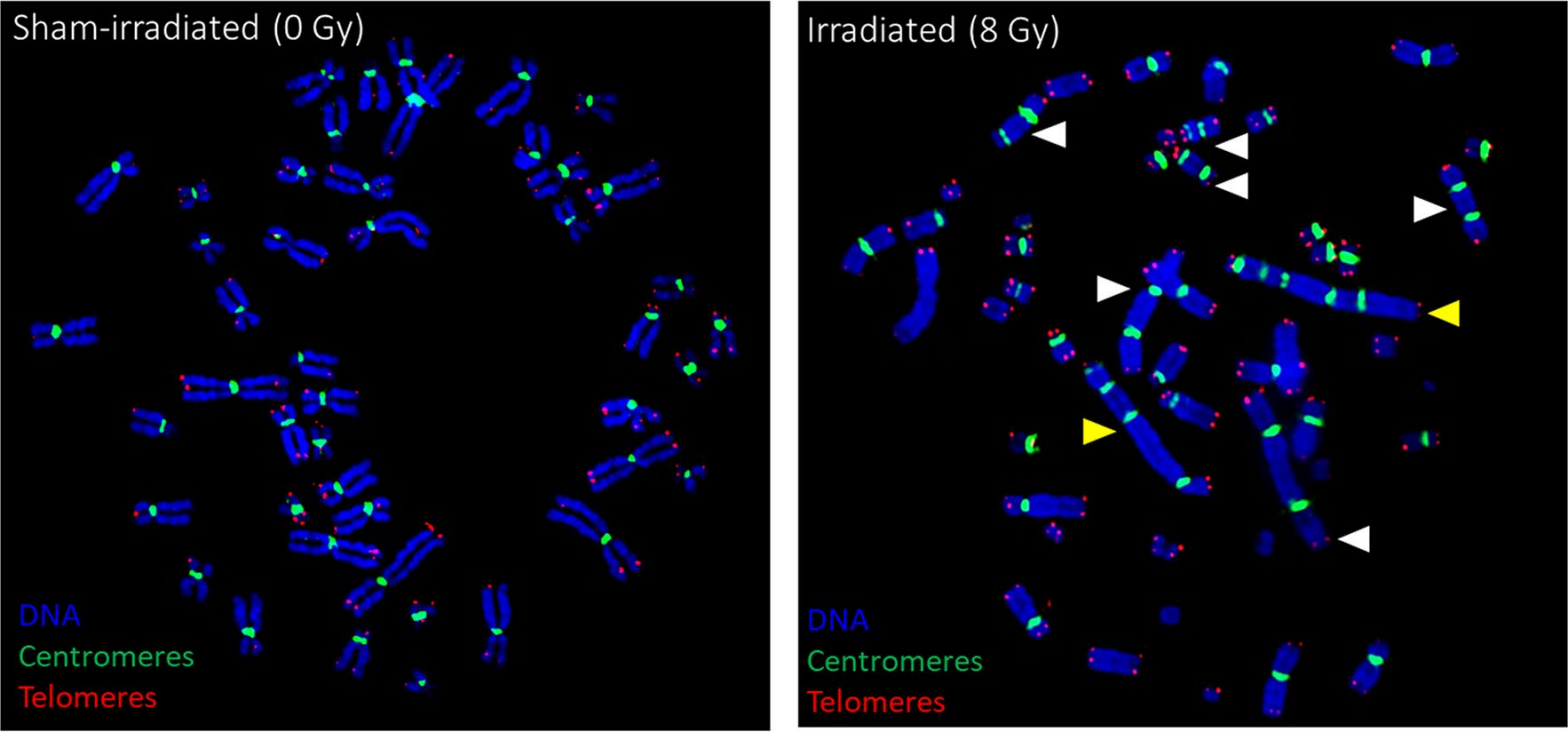
PNA-FISH-based detection of dicentric chromosomes: representative images of control (left) or irradiated to 8 Gy (right) lymphocytes. Aberant chromosomes are indicated by white (dicentrics, centromere number = 2) or yellow (multicentrics, centromere numbers > 2) arrows.

At 8 Gy, the aberrant chromosomes displayed tendencies to form very long multicentrics, with 4, 5, and 6 centromeres per chromosome (Fig. 13 and Notes to Table S1). Dicentrics were distributed unevenly: at 8 Gy 9 MeV electrons resulted in approximately –14 dicentrics/cell. At higher dose rates (5 Gy/s and 50 Gy/s), the dicentric yields reduced, however, multicentrics were still predominant over dicentrics (notes to Table S1). When the dose rate increased up to “600 Gy/s” (dose delivered in 3 pulses), the total numbers of dicentrics decreased even more, and aberrant chromosomes became shorter (mostly 2 centromeres per chromosome).

In general, the samples were in good agreement with the Poisson distribution, with only three samples where u exceeded 1.96. Negative u-values, indicating underdispersion, were recorded for the samples exposed to 3 Gy (u = − 2.33) and 8 Gy (u = − 2.46) at a dose rate of 5 Gy/s. The underdispersion could be due to a small number of donors and scored cells. The u-value was positive (u = 2.4) for 3 Gy sample exposed at a dose rate of 50 Gy/s, implying overdispersion. Dicentric overdispersion was reported earlier for radiotherapy patients subjected to higher dose rate exposures, suggesting that the proportion of undamaged lymphocytes could increase with the dose rate^35^. However, higher dose rates may not be the reason that caused overdispersion in our experiment because we did not observe the same effect for 8 Gy sample exposed at a same dose rate of 50 Gy/s.

The relationships between dose and dicentric yield at different dose rates were analyzed using the function $Y=\alpha D+\beta {D}^{2}$ (Fig. S3). A representation of this function is that dicentrics require two lesions for their formation, which are induced by one or two tracks. The α and β values shown in Table 2 indicate that α coefficients were similar (due to high errors), while the differences in the observed dicentric yields at higher dose rates were almost entirely due to the marked decrease in β coefficients (and small errors). This supports the consideration, that only β coefficient is modulated by the dose rate changes^36^. As the values α/β are 0.4 Gy and 2.6 Gy, 1.8 Gy, or 8.7 Gy for dicentrics produced at conventional (1 Gy/min) vs. higher dose rates. This may indicate that sub-lethal damage is modulated to a much higher degree than lethal damage, at high dose rates and thus dicentrics produced by single tracks will predominate.

These results are consistent with some previously reported studies. For example, Fouillade et al.^37^ have shown that, for some biomarkers, the response of normal cells to FLASH radiotherapy was different from conventional dose rate even at oxygen levels of 21%, while cancer cells were indeed insensitive to dose rate in normal air (cf. Fig. 1B in^37^). Specifically, normal fibroblasts (MRC-5 and IMR-90), irradiated in equilibrium with room air showed fewer 53BP1 foci at high dose rate compared to conventional. Cancer cells (A549), however, had similar number of foci as conventional and FLASH dose rates^36,38^.

In lymphocytes, Cooper et al.^39^ saw no difference in comet formation between FLASH and conventional dose rate, however, in their experiments, lymphocytes were analyzed immediately after irradiation. Their model may therefore lack some live cell features that may lead to a “FLASH effect” (e.g., oxygen metabolism and DNA damage processing), compared to our experimental setup, where cells were allowed to perform DNA repair for 48 h in culture. In addition, as alkaline comet assay reflects a mixture of 3 types of DNA damage (SSB, DSB, and alkaline-labile sites), it is partially difficult to extrapolate it to dicentrics data which shows repair efficacy of one type of DNA damage only (DSB).

Our data and that of others^36–39^, may indeed imply that, in contrast to cancer cells, actively metabolizing normal cells may respond differently to FLASH radiotherapy irrespectively of the oxygen levels. However, at this point, it is difficult to assume the exact mechanism that underlies these discrepancies between normal and cancer cells on the molecular level. It is also interesting, that a parallel can be drawn between our data and the data of^37,39^: in 21% O_2_, normal cells reacted differently to FLASH vs. conventional dose rate radiotherapy in DSB-related damage (53BP1 and dicentrics), but not mixed type damage (alkaline comet and H2AX assays). Probably, the assays that measure the DSB-related damage only can be more sensitive when accessing FLASH effects, while cellular assays that are less specific to the particular type of the DNA damage, to some extent, may hinder these differences in normal cells. Alternatively, we do not exclude the possibility that more experiments are needed to make solid conclusions, as to date, the experimental data accumulated by many other researchers still looks contradictive.

Experiments with a large cohort of volunteers^40^ is under way to investigate age and sex effects on the dose rate response of dicentric yields, using (a) high dose rates, using this platform, and (b) very low dose rates, mimicking fallout^12^.

## Conclusions

We have developed a high dose rate irradiator based on a retired Varian Clinac 2100C. It has been in operation for almost 2 years performing both radiation oncology-related studies at average dose rates of up to 100 Gy/s and biodosimetry-related studies at dose rates of up to 3 Gy/pulse.

In these investigations we have seen a marked reduction in dicentric yields in ex vivo irradiated blood at high dose rates.

## Supplementary Information

Supplementary Information.

## Data Availability

All data generated or analyzed during this study are included in this published article and its supplementary information files.
