# Supplementary material for: Ultra-high dose rate FLASH irradiator at the radiological research accelerator facility
Source: Sci Rep. 2022 Dec 22;12:22149. doi: 10.1038/s41598-022-19211-7 (PMC9780319; doi:10.1038/s41598-022-19211-7)
Supplement: Supplementary file 1 — Supplementary Information. [file 41598_2022_19211_MOESM1_ESM.docx]

# Supplementary Data


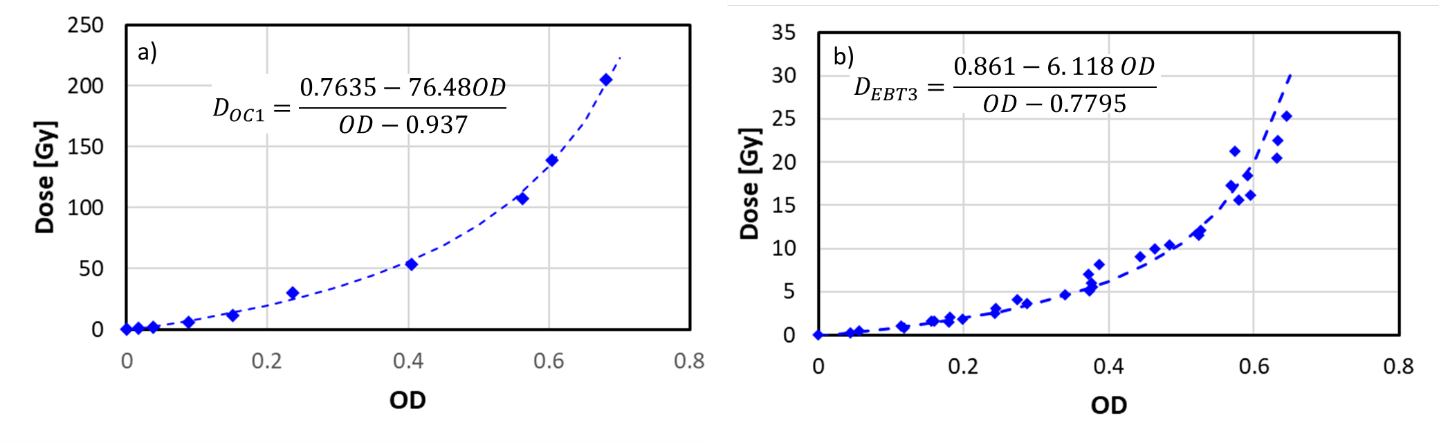


Figure S1: Calibration curves for the a) OC1 and b)EBT3 films.


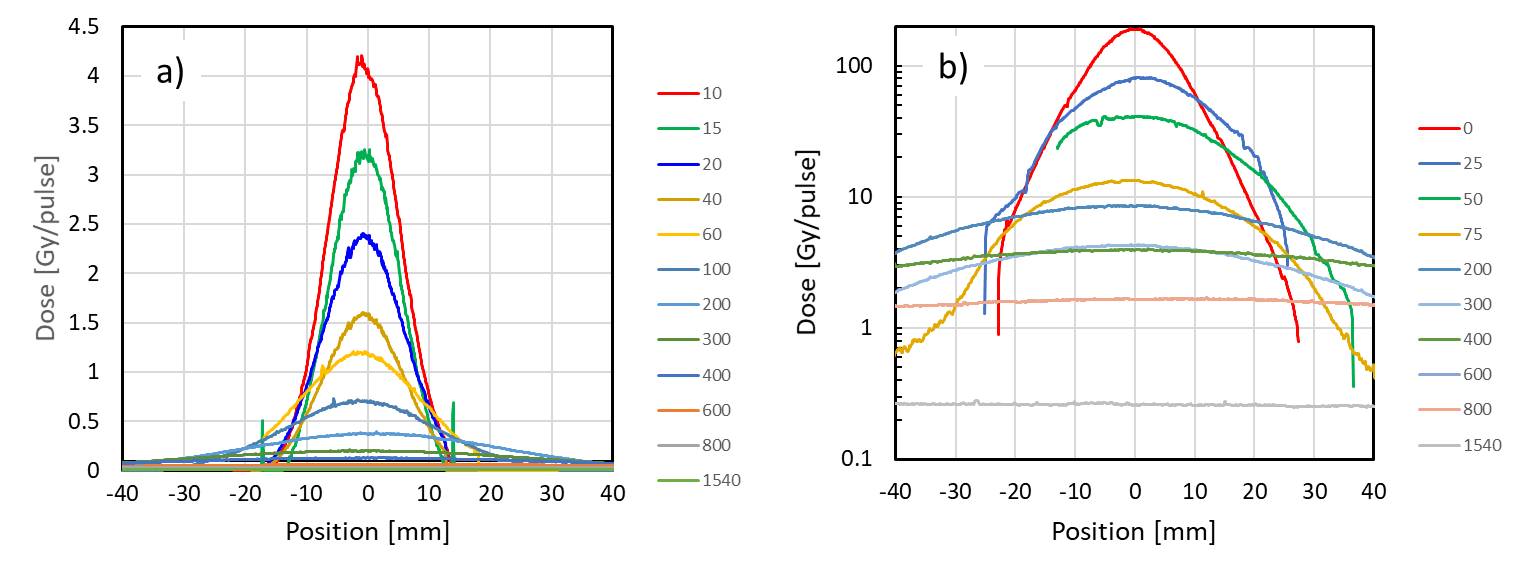


Figure S2: dose distribution at different heights for a) 9 meV beam and b) 6 MeV beam.


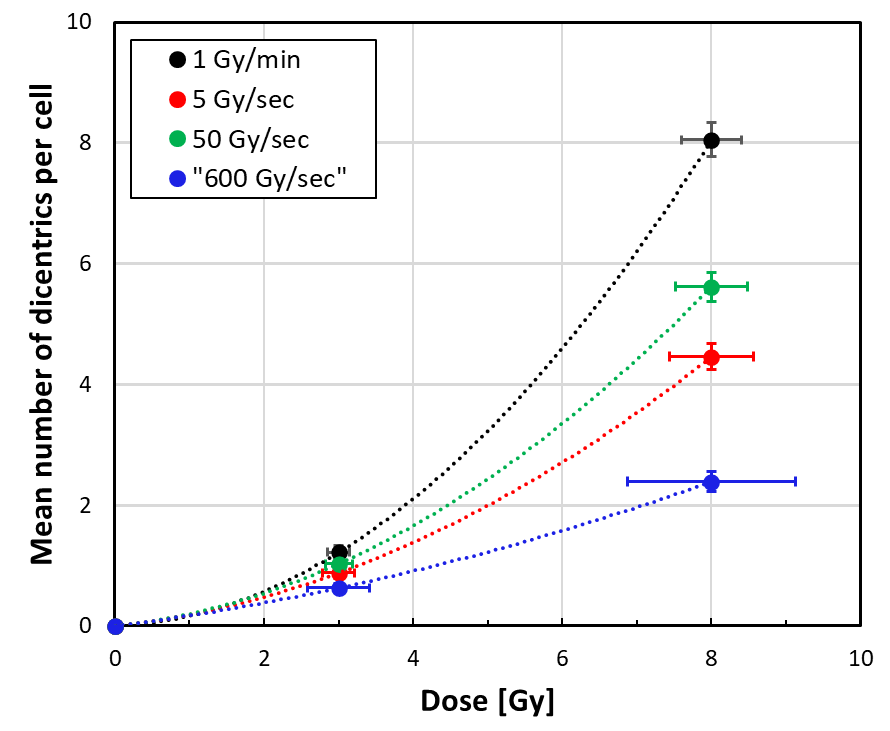


Figure S3: Dose-response curves for different dose rates (plotted using supplementary table 1).

Table S1. Yields and Intercellular Distribution of Dicentrics for Different Dose-Rates

| **Dose (Gy)** | **Dose rate** | **Cells** |  | **Distribution of dicentrics** | | | | | | | | | | | | | | | ***Y ± SE*** | ***σ^2^/y*** | ***u test*** |
| --- | --- | --- | --- | --- | --- | --- | --- | --- | --- | --- | --- | --- | --- | --- | --- | --- | --- | --- | --- | --- | --- |
|  |  |  | **DC** | **D0** | **D1** | **D2** | **D3** | **D4** | **D5** | **D6** | **D7** | **D8** | **D9** | **D10** | **D11** | **D12** | **D13** | **D14** |  |  |  |
| 0 |  | 100 | 0 | 100 | 0 | 0 | 0 | 0 | 0 | 0 | 0 | 0 | 0 | 0 | 0 | 0 | 0 | 0 | 0.00 ± 0.00 |  |  |
| 3.0 | 1 Gy/min | 100 | 122^a^ | 27 | 41 | 18 | 11 | 3 | 0 | 0 | 0 | 0 | 0 | 0 | 0 | 0 | 0 | 0 | 1.22 ± 0.11 | 0.92 | -0.57 |
|  | 5 Gy/sec | 100 | 88 | 32 | 51 | 15 | 1 | 1 | 0 | 0 | 0 | 0 | 0 | 0 | 0 | 0 | 0 | 0 | 0.88 ± 0.09 | 0.67 | -2.33 |
|  | 50 Gy/sec | 100 | 103 | 46 | 23 | 16 | 12 | 3 | 0 | 0 | 0 | 0 | 0 | 0 | 0 | 0 | 0 | 0 | 1.03 ± 0.10 | 1.34 | 2.4 |
|  | 600 Gy/sec | 100 | 63 | 58 | 25 | 13 | 4 | 0 | 0 | 0 | 0 | 0 | 0 | 0 | 0 | 0 | 0 | 0 | 0.63 ± 0.08 | 1.18 | 1.28 |
|  | 3 x 10^5^ Gy/sec | 100 | 78 | 47 | 34 | 14 | 4 | 1 | 0 | 0 | 0 | 0 | 0 | 0 | 0 | 0 | 0 | 0 | 0.78 ± 0.09 | 1.05 | 0.35 |
| 8.0 | 1 Gy/min | 100 | 805^b^ | 0 | 0 | 0 | 2 | 11 | 10 | 8 | 12 | 12 | 11 | 16 | 5 | 8 | 3 | 2 | 8.05 ± 0.28 | 0.95 | -0.35 |
|  | 5 Gy/sec | 100 | 446^c^ | 0 | 0 | 11 | 22 | 22 | 21 | 11 | 7 | 4 | 2 | 0 | 0 | 0 | 0 | 0 | 4.46 ± 0.21 | 0.65 | -2.46 |
|  | 50 Gy/sec | 100 | 562^d^ | 0 | 0 | 5 | 12 | 16 | 17 | 18 | 15 | 8 | 4 | 2 | 2 | 1 | 0 | 0 | 5.62 ± 0.24 | 0.82 | -1.27 |
|  | 600 Gy/sec | 100 | 239^e^ | 3 | 30 | 27 | 17 | 16 | 4 | 2 | 0 | 1 | 0 | 0 | 0 | 0 | 0 | 0 | 2.39 ± 0.16 | 0.90 | -0.71 |

***Notes:***

DC: dicentric chromosomes; Y ± SE: yield ± standard error, calculated using Dose Estimate software[21]; σ^2^/y: dispersion index (σ: variance, y: mean); u: normalized unit of the dispersion index

Recorded multicentric aberrations before conversion to dicentrics:

*^a^ 1 tricentric*

*^b^ 83 tricentrics, 10 tetracentrics, 2 pentacentrics*

*^c^ 30 tricentrics, 2 tetracentrics*

*^d^ 31 tricentrics, 7 tetracentrics, 1 pentacentric, 1 heptacentric*

*^e^ 7 tricentrics*
